# Purification and Characterization of a Novel Hypersensitive Response-Inducing Elicitor from *Magnaporthe oryzae* that Triggers Defense Response in Rice

**DOI:** 10.1371/journal.pone.0037654

**Published:** 2012-05-18

**Authors:** Mingjia Chen, Hongmei Zeng, Dewen Qiu, Lihua Guo, Xiufen Yang, Huaixing Shi, Tingting Zhou, Jing Zhao

**Affiliations:** Key Laboratory of Integrated Pest Management in Crops, Ministry of Agriculture, Institute of Plant Protection, Chinses Academy of Agricultural Sciences, Beijing, China; Seoul National University, Republic of Korea

## Abstract

**Background:**

*Magnaporthe oryzae*, the rice blast fungus, might secrete certain proteins related to plant-fungal pathogen interactions.

**Methodology/Principal Findings:**

In this study, we report the purification, characterization, and gene cloning of a novel hypersensitive response-inducing protein elicitor (MoHrip1) secreted by *M. oryzae*. The protein fraction was purified and identified by de novo sequencing, and the sequence matched the genomic sequence of a putative protein from *M. oryzae* strain 70-15 (GenBank accession No. XP_366602.1). The elicitor-encoding gene *mohrip1* was isolated; it consisted of a 429 bp cDNA, which encodes a polypeptide of 142 amino acids with a molecular weight of 14.322 kDa and a pI of 4.53. The deduced protein, MoHrip1, was expressed in *E. coli*. And the expression protein collected from bacterium also forms necrotic lesions in tobacco. MoHrip1 could induce the early events of the defense response, including hydrogen peroxide production, callose deposition, and alkalization of the extracellular medium, in tobacco. Moreover, MoHrip1-treated rice seedlings possessed significantly enhanced systemic resistance to *M. oryzae* compared to the control seedlings. The real-time PCR results indicated that the expression of some pathogenesis-related genes and genes involved in signal transduction could also be induced by MoHrip1.

**Conclusion/Significance:**

The results demonstrate that MoHrip1 triggers defense responses in rice and could be used for controlling rice blast disease.

## Introduction

In nature, plants face a broad range of potential pathogens, including viruses, bacteria, fungi, oomycetes, nematodes and insects, which have different life cycles and infection strategies. However, only some of these potential pathogens can actually establish an interaction with a plant by successfully recognizing and conquering the host plant's defense system. Plants rely on two types of basal defenses to prevent infection by pathogens [1], [2], [3]. One type uses a recognition system that identifies microbial- or pathogen-associated molecular patterns (MAMPs or PAMPs) [4], such as flagellin, to prevent infection by pathogens. This innate immunity of the plants triggered by PAMP via several plant transmembrane pattern recognition receptors (PRRs) is called PAMP-triggered immunity (PTI). The other type of defense, named gene-for-gene resistance, acts mainly inside the plant cell, using the recognition between the resistance (R) proteins and the effectors, including elicitors secreted by pathogens [5]. This R protein-mediated defense is called effector-triggered immunity (ETI); it occurs only in living host tissues but not in necrotrophs [6], [7] and accompanies some early events, such as the hypersensitive response (HR), oxidative bursts, nitric oxide (NO) generation, extracellular pH increase, cell wall strengthening and expression of pathogenesis-related proteins [8], [9], [10], in plant cells. Moreover, these resistance responses first occur at the site of infection in the cells and subsequently extend to neighboring and non-infected cells, eventually creating systemic acquired resistance (SAR) in whole plants to fight effectively against various kinds of pathogens [11], [12].

Many elicitors have been isolated from various organisms, including bacteria, viruses, oomycete and fungi. The elicitor molecules have included proteins, peptides, glycoproteins, lipids and oligosaccharides [13], [14], [15], and some of these elicitors have been used to improve the pathogen resistance of plants [16]. These defense responses are often associated with HR and even localized programmed cell death [17], involving ion influx, NO production and reactive oxygen species (ROS), such as H_2_O_2_ and O_2_^−^, which then transduce the elicitor signal to downstream defense responses as signaling molecules [18]. In the whole-plant defense system, ROS are thought to play a key function in the elicitor signal transduction system and are also correlated with the HR and cause general cell death [19]. In addition, hydrogen peroxide, as a ROS and a typical second messenger, directly and indirectly regulates the plant downstream innate immune system [20], inducing the production of secondary metabolites, such as phytoalexins and antifeedants, to supplement the plant defense system [21].

*Magnaporthe oryzae*, known as the rice blast fungus [22], is a plant-pathogenic fungus that causes economically significant crop losses annually [23]. Some elicitors have been isolated from *M. oryzae*, including two glycoproteins that have been shown to induce resistance responses in rice [24], [25], two sphingolipid elicitors that have been shown to induce hypersensitive cell death and phytoalexin accumulation in rice [26] and a protein elicitor that has been shown to induce SAR in plants [27], [28]. All of these elicitors could boost the resistance of the plant to the pathogen, suggesting a potential pathway for fighting rice blast. However, none of the elicitors mentioned above is a secreted protein. *M. oryzae* might also secrete some proteins related to the plant-fungus interaction [29].

In this article, we first report the purification and characterization of a novel secreted protein elicitor from *M. oryzae*. This protein could induce the early signaling events of plant defenses in tobacco plants and enhance the basal defense of rice seedlings against infection by *M. oryzae*. Our research helps to elucidate the early interactions between plants and pathogens and may provide a novel strategy for controlling plant disease [30], [31].

## Results

### Purification and characterization of the MoHrip1 protein

**Figure 1:**
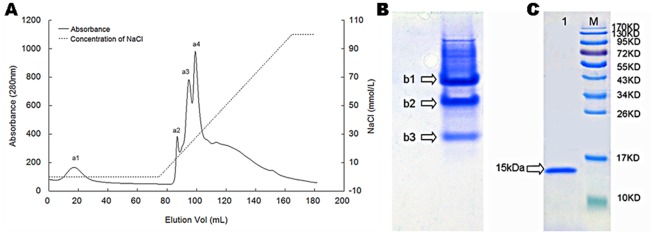
The purification of MoHrip1 from *M. oryzae*. **A**. Concentrated culture filtrate was loaded on HiTrap™ Q HP 5 ml column at a flow rate of 2 ml/min. Four peaks were collected, and the target protein was included in peak a2. **B**. Peak a2 was pooled and applied to native-PAGE and electroelution. The b3 fraction was the target elicitor protein. **C**. SDS-PAGE analysis of the purified elicitor protein, MoHrip1, showing a single band with Coomassie Brilliant Blue R-250 staining. (*1*: MoHrip1, *M*: protein molecular weight marker).

Crude protein from a culture filtrate of *M. oryzae* was put through dialysis and anion exchange chromatography, and final extracts were eluted with a NaOH gradient. All peaks were collected and infiltrated into tobacco leaves to test for the HR (data not shown), and the concentrated fraction of peak a2 (Figure 1A) was selected for further purification due to the presence of HR activity. Through further purification with cutting native-PAGE and electroelution, protein b3 (Figure 1B) was isolated. This protein showed strong HR activity, displayed a single band on SDS-PAGE and a single spot by two-dimensional gel electrophoresis (2-DE) (Figure S1) and had a relative apparent molecular weight of 15 kDa (Figure 1C). We designated this protein *Magnaporthe oryzae* hypersensitive response-inducing protein 1 (MoHrip1).

**Figure 2:**
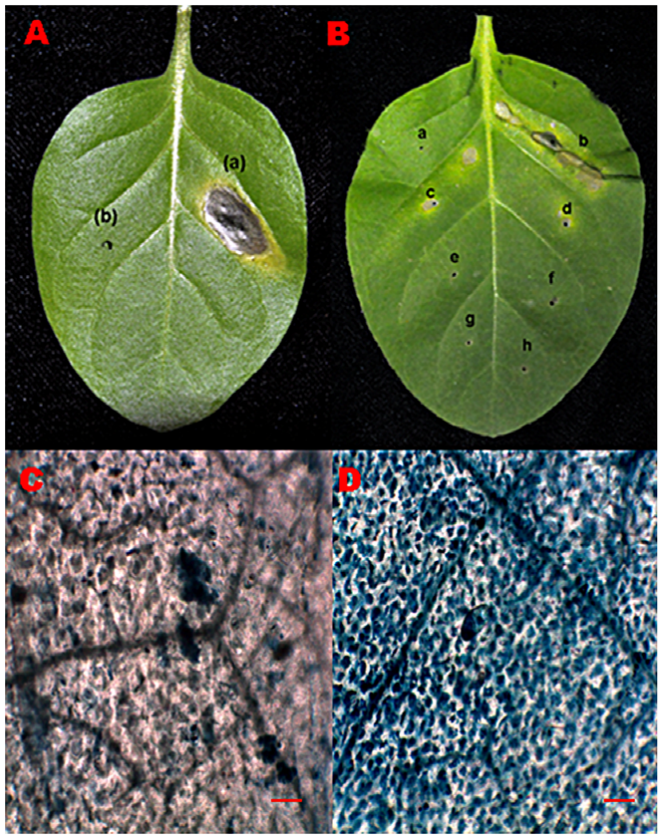
The hypersensitive response induced by MoHrip1 in tobacco leaves. **A**. The response was photographed 24 hours after injection. (a) The MoHrip1 treatment (30 µM), (b) Control treatment with Mes-NaOH buffer (20 mM). **B**. Series of concentration-induced HR activity. a: 30 µM Bovine serum albumin (BSA) b-h: 30 µM, 10 µM, 5 µM, 1 µM, 0.5 µM, 0.1 µM and 0.01 µM MoHrip1, respectively. The elicitor protein was dissolved in sterile distilled water in series of concentrations. The minimum concentration of MoHrip1 that induced HR activity was 5 µM. C. Tobacco leaves with control treatment (20 mM) stained by trypan blue. D. Tobacco leaves with MoHrip1 treatment (30 µM) stained by trypan blue. Dead tobacco leaf cells, which are the hallmark of HR, were stained by trypan blue. Scale bar = 10 µM.

Infiltration of MoHrip1 into mature tobacco leaves resulted in rapid macroscopic changes. HR induced by the purified elicitor protein was determined through observing the development of the necrosis spot at the site of injection in tobacco leaves. The infiltrated area of tissue became obviously transparent 8–10 h after inoculation and turned into a necrotic lesion 20–24 h after treatment (Figure 2A). Serial dilution of MoHrip1 tested for HR indicated that the minimum concentration of the elicitor that induced a HR was 5 µM (Figure 2B). This concentration of the elicitor was used in the subsequent experiments. HR was also monitored base on observing dead cells after staining leaves with trypan blue (Figure 2C and 2D). Dead tobacco leaf cells, located in the site of HR, were stained in blue (Figure 2D).

The pI of the MoHrip1 was determined by 2-DE, and the protein resolved as a single spot in the acidic region with relative pI of 4.53 (Figure S1). MoHrip1 was shown to be a pure protein with no carbohydrate moieties (Figure S2). The thermostability experiment indicated that MoHrip1 was stable at 4 or 25°C for 15 min and retained its biological activity of inducing a HR, but the protein was denatured at 60, 80 and 100°C (data not shown). MoHrip1 also lost its HR activity after treatment with proteinase K solution (10 µg/ml).

### Amino acid sequencing and cloning of MoHrip1

The protein spot was excised from the 2-DE gel (Figure S1) for liquid chromatography-mass spectrometry analysis of in-gel digested proteins for amino acid sequence determination of MoHrip1. A series of unique polypeptides were measured by matrix-assisted laser desorption/ionization time-of-flight (MALDI-TOF) and analyzed by de novo sequencing. Three peptide segments with reliable amino acid sequences were obtained: ^40^TVDNTPTNVNFK^51^, ^76^KCGDSAYSFAIV^87^ and ^102^GPGVGLYGQG^112^. Similarity matching by protein BLAST search indicated that the protein presented high identity to a conserved hypothetical protein (GenBank accession number: XP_366602) from the *M. oryzae* strain 70-15. Using the sequence of strain 70-15, PCR primers were designed to clone the gene encoding MoHrip1.

A full-length cDNA encoding MoHrip1 was amplified from *M. oryzae* (GenBank accession number: JQ231215) by reverse transcription-polymerase chain reaction (RT-PCR). The *mohrip1* gene includes an open reading frame of 429 bp encoding a protein of 142 amino acids with a theoretical molecular weight of 14.322 kDa. Analysis of the deduced amino acid sequence of MoHrip1 (Blastp) indicated that MoHrip1 shares high similarity to proteins from several plant fungal pathogens. The highest levels of similarity were 99% and 97% to the hypothetical proteins GLRG_08187 (GenBank accession No. EFQ33043.1) and GLRG_07615 (GenBank accession No. EFQ32601.1) from *Glomerella graminicola* M1.001, respectively. The deduced amino acid sequence also showed 79% identity to hypothetical protein FG03035.1 (GenBank accession No. XP 383211.1) from *Gibberella zeae* PH-1 and 78% identity to protein elicitor PevD1 (GenBank accession No. ADW79419.1) from *Verticillium dahliae*.

Analysis of the MoHrip1 cDNA sequence using the SignalP 4.0 Server (http://www.cbs.dtu.dk/services/SignalP/) revealed that it contains a 16 amino acid signal peptide (Figure S3), indicating that MoHrip1 is a secreted protein.

### Heterologous expression and purification of the elicitor protein

**Figure 3:**
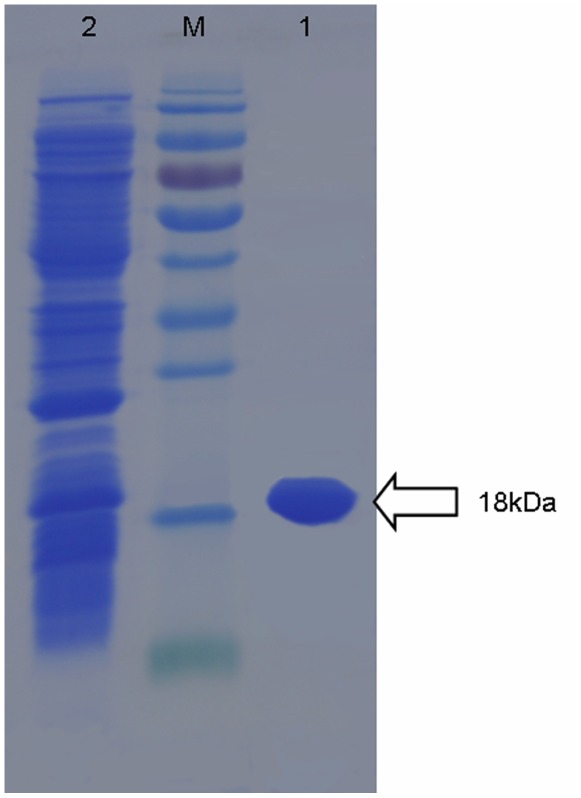
Purification of recombinant MoHrip1. *M*: protein molecular weight marker, *1*: Purified His-tagged MoHrip1, *2*: Total *E. coli* expressed proteins.

The *mohrip1* gene was cloned into a pET-30a expression vector with a His_6_ tag and subsequently transformed into *E. coli* transetta (DE3) competent cells. The prokaryotically expressed His_6_-MoHrip1 was soluble in *E. coli*, and we sequentially purified it. The purified recombinant protein exhibited a single band with an apparent molecular mass of approximately 18 kDa on SDS-PAGE (Figure 3). The typical HR in tobacco leaves could also be induced by the purified recombinant elicitor protein (data not shown).

### Early events induced by the elicitor MoHrip1 in tobacco

**Figure 4:**
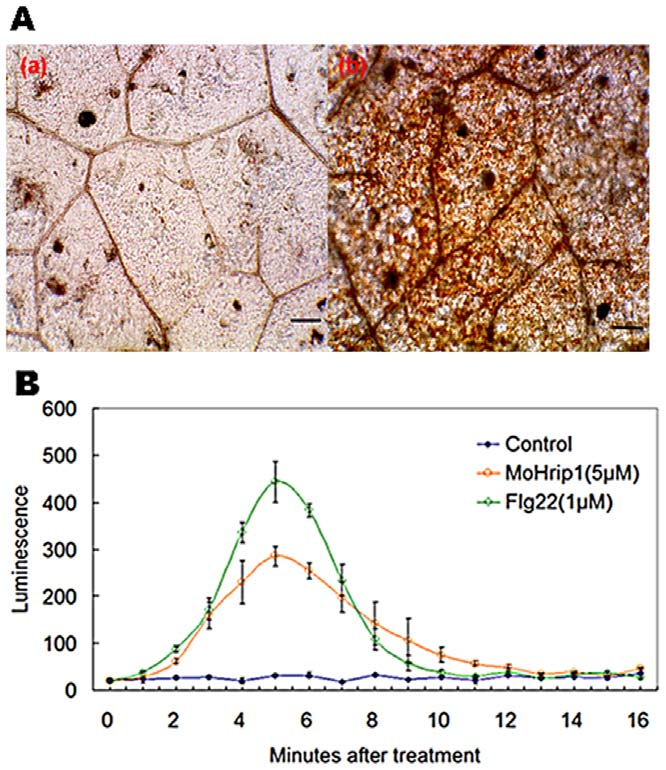
ROS burst in tobacco after MoHrip1 treatment. **A**. Microscopic observation of H_2_O_2_ accumulation in tobacco leaves. (a) Tobacco leaves treated with sterile distilled water, (b) MoHrip1-treated leaves. H_2_O_2_ accumulation (as indicated by diaminobenzidine staining) appeared in the veins and stomata of elicitor-treated leaves but not in leaves treated with sterile distilled water. Scale bar = 50 µm. **B**. ROS formation in tobacco cell culture after elicitor treatment, flg22 treatment and sterile distilled water treatment was detected in 96-well plates by chemiluminescence. ROS formation in both the MoHrip1-treated and flg22-treated cell cultures reached a maximum at approximately 5 min and declined thereafter to the level of the negative control. Each data point represents three replicates. Error bars represent ± SD of the mean.

A burst of oxidative metabolism leading to the generation of superoxide (O_2_^−^) and subsequent accumulation of hydrogen peroxide (H_2_O_2_) is one of the earliest significant events before the HR [32]. The MoHrip1 protein induced H_2_O_2_ accumulation in tobacco leaves (Figure 4A) and caused a ROS burst in tobacco cell culture (Figure 4B). H_2_O_2_ was detected by 3,3′-diaminobenzidine (DAB) staining, and sites of H_2_O_2_ accumulation were clearly observed in the veins and stomata of tobacco leaves under the microscope (Figure 4A). By chemiluminescence, ROS production induced by the MoHrip1 elicitor, which was dissolved in sterile distilled water, was determined in tobacco suspension and compared to an flg22 treatment positive control and a sterile distilled water treatment negative control (Figure 4B). MoHrip1 treatment caused a rapid increase in H_2_O_2_, which was detected immediately after application and reached a maximum at approximately 5 min. Subsequently, the H_2_O_2_ level gradually decreased to approximately that of the negative control.

**Figure 5:**
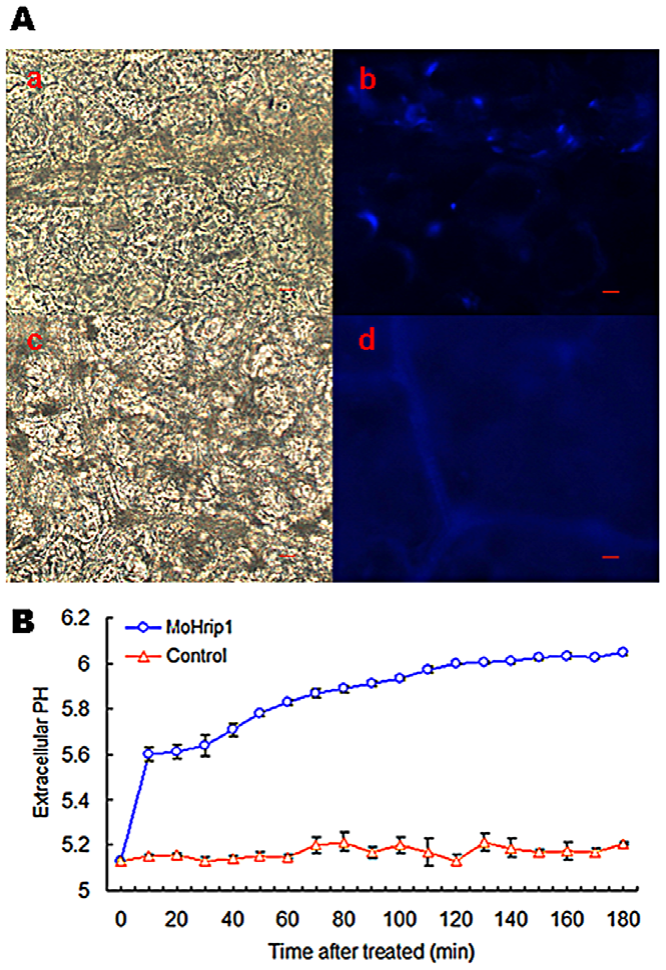
MoHrip1-induced callose deposition and extracellular medium alkalinization in tobacco. **A**. Callose deposition in tobacco leaves. Tobacco leaves were infiltrated with MoHrip1 (50 µL of a 5 µM solution of MoHrip1) or Mes-NaOH buffer (20 mM, pH 6.0) as a control. After a 24 hour incubation period, the tobacco leaves were bleached to remove their chlorophyll and then stained with aniline blue. The samples were observed under bright-field (a and c) and UV fluorescence (b and d) microscopy. Apparent punctiform callose deposits around the cell wall were photographed in the MoHrip1-treated leaves (b). a and b: MoHrip1-treated leaves, c and d: control. Scale bar = 10 µM. **B**. The kinetics of the extracellular medium alkalinization induced by MoHrip1 in tobacco suspension. A distinct pH increase in the elicitor-treated cell culture was monitored for the first 10 minutes, and the pH stabilized after 100 minutes. Each data point represents three replicates. The error bars represent ± SD of the mean.

In most of plant tissues, H_2_O_2_ is a key signal molecule, which could induce arrays of cellular protectant and defense genes and cause the callose deposition of challenged cells [32], [33], [34]. Therefore, we tested whether the MoHrip1 elicitor induced the deposition of callose. We could easily observe the typical necrotic lesions by staining with aniline blue at the injection site of the tobacco leaves 24 h after infiltration with MoHrip1. A punctiform pattern of callose deposition was detected at the mesophyll cell walls in the necrotic area, and the control buffer (Mes-NaOH) treatment had no effect on callose deposition in tobacco leaves (Figure 5A).

As another typical early event after challenging a cell suspension with an elicitor, alkalinization of the tobacco cell culture medium caused by MoHrip1 was also analyzed. The pH of the tobacco cell suspension increased significantly compared to that of the control within 10 minutes after treatment with the elicitor (Figure 5B).

### Induction of resistance and ROS to rice blast by MoHrip1

**Figure 6:**
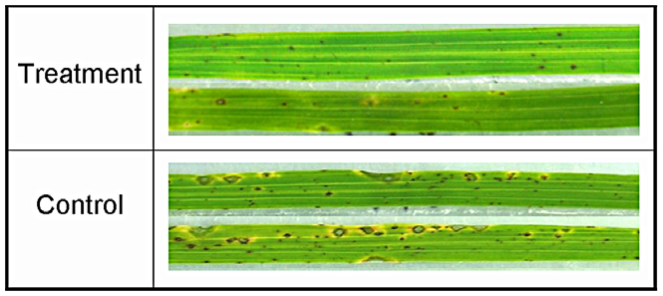
Representative disease symptoms on leaves of the MoHrip1-treated and control rice seedlings. Rice seedlings were sprayed with MoHrip1 (5 µM) or Mes-NaOH (20 mM) as a control 3 days before being treated with *M. oryzae* spores. The leaves of representative plants were photographed 7 days post-inoculation.

Groups of soil-grown rice plants were sprayed with a *M. oryzae* spore suspension (strain RO1-1) after 3 days of treatment with either the elicitor or a control solution (elicitor buffer, Mes-NaOH). Screening was carried out on detached leaves. Control plants exhibited clear symptoms at almost 4 days post-inoculation, but no blast disease symptoms were observed on the leaves of plants treated with MoHrip1 at this time. Seven days after inoculation, the lesions on the leaves of control plants dramatically increased in size, and the rice blast symptoms were typical and severe. At this time, only small and constrained lesions were observed on the leaves of MoHrip1-treated plants, and most of the plants remained green and healthy (Figure 6). The disease severity of all plants from both the control and elicitor treatments was evaluated on a standard international 0–9 scale at 7 days and 15 days post-inoculation, and the leaf blast score of MoHrip1 treatment seedlings was much lower than that of control plants (Table 1). At 15 days post-inoculation, the control plants were severely damaged and even withering. At this time, only small lesions were generally observed on the leaves of MoHrip1-treated plants, and the plants were still alive (data not shown). Taken together, the results from the disease-resistance assays demonstrated that MoHrip1 could increase rice plant resistance to the blast fungus *M. oryzae*.

**Table 1:** Disease severity of rice blast in leaves of elicitor-treated and control-treated rice plants.

	Rice blast score	
Rice seedling samples	7 days post-inoculation	15 days post-inoculation
MoHrip1 treatment	2.646±0.355A	2.854±0.425A
CK treatment	5.646±0.237B*	5.792±0.201B*

Soil-grown rice seedlings were sprayed with *M. oryzae* spores. The disease scores of the rice seedlings were evaluated on a scale of 0–9 at 7 and 15 days post-inoculation.

* Data are representative of three replicates and sixteen plants per replicate. Values are the means±standard deviation. Within columns, values with different letters are significantly different at the 1% significance level.

**Figure 7:**
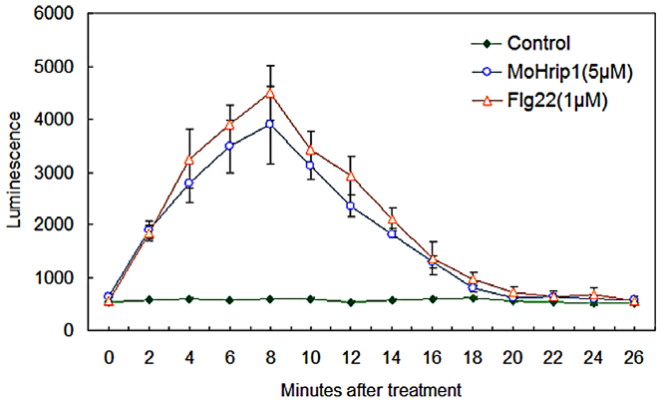
ROS generation induced by MoHrip1 in rice. A H_2_O_2_ burst was obviously elicited by MoHrip1 (dissolved in sterile distilled water) or flg22 (positive control, dissolved in sterile distilled water) within 10 minutes in rice leaves, but no H_2_O_2_ burst was detected in the samples treated with sterile distilled water (negative control). After 20 minutes, the level of ROS production in MoHrip1- or flg22-treated samples decreased to that in the negative control samples. The error bars represent ± SD of the mean. Essentially identical results were obtained in three independent experiments.

The oxidative burst has been implicated in cellular defense responses [35], and ROS are directly protective and drive oxidative cross-linking of the cell wall. Using chemiluminescence, we evaluated the ROS production in MoHrip1-treated rice leaves (Figure 7) compared to flg22 as a positive control. This data indicated that MoHrip1 could induce ROS production in rice leaves within 10 minutes as well as flg22, suggesting that MoHrip1 could trigger the defense network of rice against pathogenic fungus infection through inducing ROS production.

### Expression of pathogenesis-related genes induced by MoHrip1

**Figure 8:**
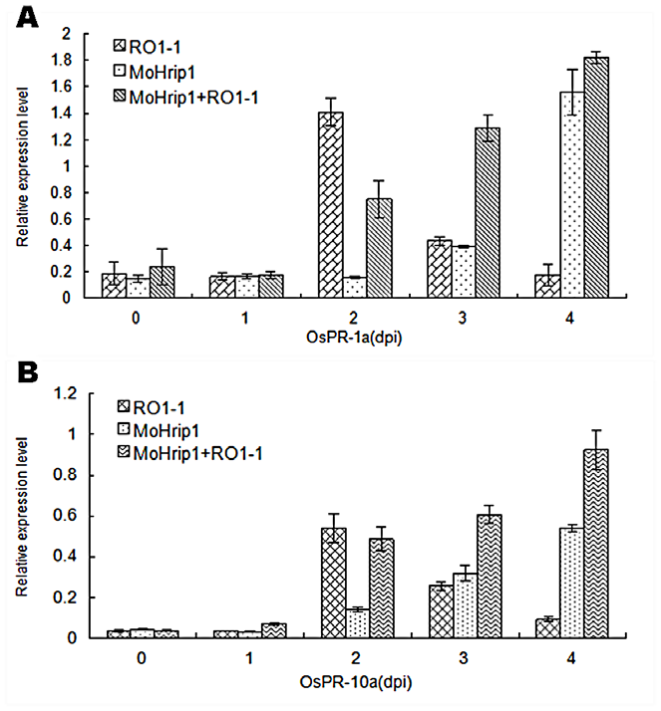
Defense-related gene expression induced by MoHrip1 in rice leaves. PR genes were induced and their expression persisted for days in *M. oryzae* strain RO1-1-treated plants and in MoHrip1-pretreated plants that were not exposed to *M. oryzae* strain RO1-1. In contrast, the expression of PR genes was induced after 2 days of treatment and declined thereafter in the RO1-1-treated samples. The error bars represent ± SD of the mean. Essentially identical results were obtained in three independent experiments. **A**. Expression of OsPR-1a induced by MoHrip1 in rice leaves, **B**. Expression of OsPR-10a induced by MoHrip1 in rice leaves.

To further investigate the mechanism associated with MoHrip1-induced resistance in rice, we analyzed the expression pattern of two rice pathogenesis-related (*PR*) genes: *OsPR-1a* (encoding *Oryza sativa* PR protein 1) (Figure 8A) and *OsPR-10a* (encoding *Oryza sativa* PR protein 10a) (Figure 8B), which have been frequently used as marker genes for defense responses in rice plants [36]. The expression of these two genes induced by MoHrip1 in rice leaves at 0, 1, 2, 3 and 4 days after incubation is shown in Figure 8. We also monitored the expression of these genes after inoculating with a *M. oryzae* spore suspension in rice plants and both MoHrip1- and RO1-1-treated rice plants with *M. oryzae*, respectively. The expression of the PR genes was rapidly induced in elicitor-treated plants at 3 days post-inoculation and obviously induced at 2 days post-inoculation before returning to the original levels at 4 days post-inoculation in the pathogen-incubated plants. By contrast, the RO1-1 treatment samples that had been inoculated with MoHrip1 beforehand had a high level of expression of the PR genes from 2 days post-inoculation until at last 4 days post-inoculation. These data suggest that MoHrip1 may enhance SAR against blast disease in rice seedlings through inducing the expression of PR genes.

### The defense signaling network depends on MoHrip1 in rice plants

**Figure 9:**
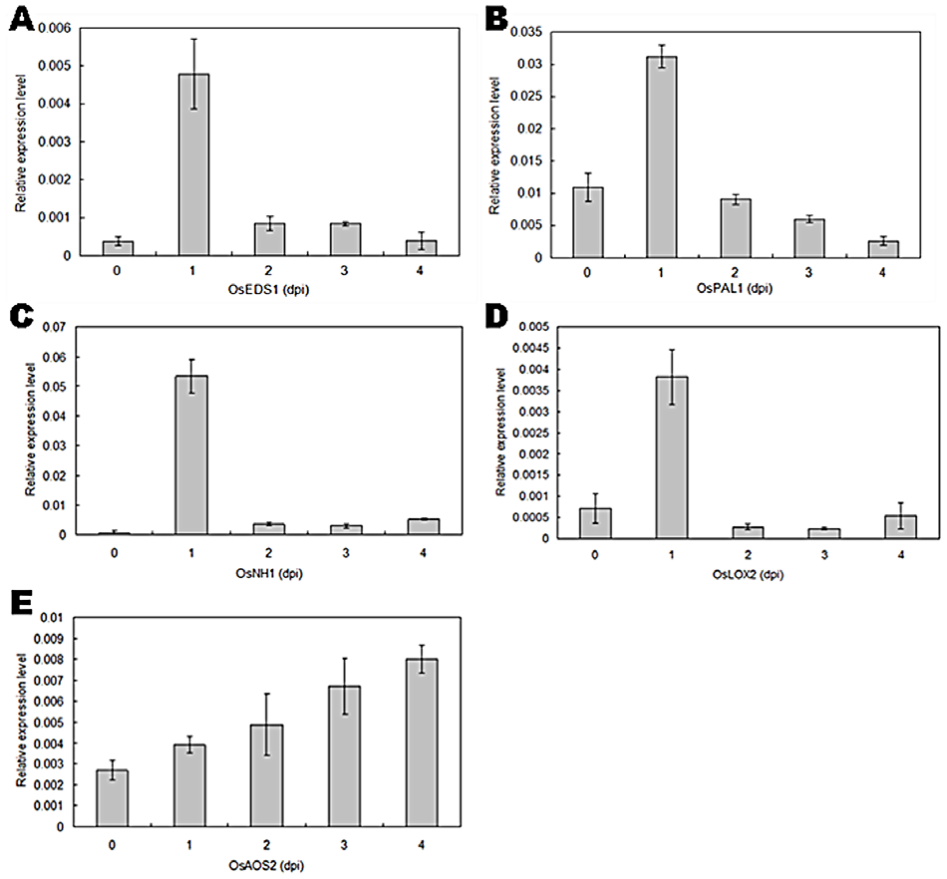
Certain marker genes of the defense signaling pathway are involved in the effects of MoHrip1 on rice. **A**. *OsEDS1*, **B**. *OsPAL1*, **C**. *OsNH1*, **D**. *OsLOX2*, **E**. *OsAOS2*. Marker genes for the SA-dependent defense pathway (*OsEDS1*, *OsPAL1* and *OsNH1*) were all significantly induced by MoHrip1 on the second day after treatment, and the same expression pattern was found for the JA/Et pathway gene *OsLOX2*, but strong induction was not observed for *OsAOS2* (another JA/Et signaling pathway marker gene). Dpi: days post-inoculation. Error bars represent ± SD of the mean. Essentially identical results were obtained in three independent experiments.

In studies, the salicylic acid (SA) signaling pathway and the jasmonic acid and ethylene (JA/Et) pathway have been described as having central importance for plant defensive responses [37], [38], [39], [40]. To study which pathways are affected by MoHrip1, the effects of MoHrip1 on the expression levels of SA- and JA/Et-dependent defense pathway genes in rice plants were analyzed using real-time quantitative PCR (Figure 9). The expression of SA signal-related genes (*OsEDS1*, *OsPAL1* and *OsNH1*) (Figure 9A–C) and a JA/Et signal-induced gene (*OsLOX2*) (Figure 9D) in rice plants was markedly induced on the second day after treatment with MoHrip1. However, the expression of another JA/Et pathway-related gene, *OsAOS2* (Figure 9E), showed almost no remarkable alteration in rice leaves that had been sprayed with MoHrip1. These results demonstrated that, in rice plants, the defense pathways related to gene expression induced by MoHrip1 might be dependent on the SA signaling pathways. Furthermore, the specific and precise signaling pathway involved in the effect of MoHrip1 on rice plants remains to be clarified by more advanced studies.

## Discussion

In this study, we purified a 14 kDa hypersensitive response-inducing protein from the culture filtrate of *M. oryzae* and cloned the gene encoding this protein. This elicitor protein, MoHrip1, can induce the transient expression of PR genes in rice and increase the resistance of rice seedlings to *M. oryzae*. Previously, we reported the isolation of some protein elicitors, such as PeaT1, PemG1 and PebC1 [27], [41], [42], from plant pathogenic fungi. All of these elicitors enhanced plant disease resistance, but none of them could induce the HR in plants. In addition, some elicitors purified from *M. oryzae* had been reported, and most of these elicitors could also induce the defense response against pathogen infection in plant seedlings [24], [25], [26], [27]. However, none of these elicitors is a secreted protein. Here, we report the first HR-inducing secreted elicitor from *M. oryzae*.

Many elicitors have been purified from plant pathogens and exhibit the ability to enhance the resistance of the plants to their pathogens, such as harpin (the first reported elicitor) [43], elicitin and cryptogein [44]. In recent years, more elicitors have been reported from pathogenic fungi. For instance, the PaNie protein elicitor isolated from *Pythium aphanidermatum* was shown to induce multiple defense responses in plants, including carrot, Arabidopsis and tobacco [45]. Two distinct elicitors, botrycin and cinerein from *Botrytis cinerea*, were able to elicit early defense responses in grapevine [46]. An elicitor derived from *Phytophthora colocasiae* enhanced SAR in taro plants and fought *Phytophthora* leaf blight [47]. A hypersensitive response-inducing elicitor, PevD1, obtained from *Verticillium dahliae* has also been shown to evoke resistance responses in tobacco [48]. All of these elicitors are secreted by the plant pathogens and could improve the ability of plants to resist pathogen infection. Furthermore, MoHrip1, the secreted protein we isolated from *M. oryzae*, had high similarity with some hypothetical proteins from plant pathogenic fungi, such as *Glomerella graminicola*, *Gibberella zeae* and *Verticillium dahliae*. Especially, the three-dimensional structure of MoHrip1 (Zhang and Chen, in preparation) shows significant similarity to that of another elicitor, PevD1 [48]; therefore, the common functional domain and molecular mechanism of both elicitors in the development of plant resistance against pathogens should be further investigated. Interestingly and conspicuously, four biotrophy-associated secreted (BAS) proteins, which are all secreted by biotrophic invasive hyphae of *M. oryzae*, are identified by the Valent lab recently [49], [50]. All the four secreted BAS proteins participate in the pathogenic process; similarly, MoHrip1 is also secreted by *M. oryzae* and could further trigger the defense pathways in rice, suggesting that MoHrip1 might be a candidate elicitor helping the pathogenic infection. However, the exact role that MoHrip1 plays in the pathogenic process is unknown, and further study is in preparation, Taken together, this information indicates that MoHrip1 is a novel elicitor protein from *M. oryzae* that can trigger the defense network of rice plants.

Plants become resistant to most of their potential invaders – plant pathogens –through a cascade of early events that lead to the induction of the plant immune system [5], [51], [52]. Many reports have demonstrated that the ROS play a crucial role in the whole-plant defense system [19], [20], and the oxidative burst often appears in the host or non-host plants after infection by a pathogenic fungus or treatment with a fungal elicitor. Various other signaling molecules involving in the plant defense response, including free calcium ions, NO, callose deposition, PAL activation, phenolics deposition and programmed cell death, are also required for elicitor-induced ROS production [18]. All of these signaling molecules could be involved in the defense response and might even control and regulate many other interconnecting downstream processes of resistant signaling pathway by metabolic changes and gene expression [8]. In this study, we mainly focus on the analysis of ROS induced by MoHrip1 in tobacco and in rice. We observed histological imaging of H_2_O_2_ in tobacco leaves and the ROS production induced by MoHrip1 in tobacco cell culture. Using an accepted elicitor, flg22, as a positive control, MoHrip1-treated and flg22-induced tobacco suspensions showed similar ROS accumulation patterns in a chemiluminescence experiment, indicating that MoHrip1 behaved like a well-known elicitor. Moreover, several other well-known early events accompanying the HR were also observed after treatment with MoHrip1, such as alkalinization of the extracellular medium, which might be due to the elicitor-induced ion fluxes, callose deposition, and NO accumulation (data not shown). All of the data indicated that MoHrip1 is a real elicitor.

As an important function, many fungal elicitors could relieve pathogen-infection damage to plants. Similarly, MoHrip1 pre-treatment could significantly alleviate the degree of rice blast infection compared to control plants, suggesting that MoHrip1 might boost the resistance of rice seedlings to the pathogen *M. oryzae*. Some plant PR genes, such as *PR-1a, PR-1b*, *PR-10a*, are involved in the plant defense network when plants encounter a pathogen infection [36], [53]. In this paper, the expression of two typical PR genes, *OsPR-1a* and *OsPR-10a*, in the elicitor-induced plants, the pathogen-infection plants and the plants that underwent both treatments was analyzed (Figure 8). Transcripts of the tested PR genes began to accumulate after 3 days in MoHrip1-treated plants. Although pathogen infection induced the expression of these genes at 2 days after treatment, the effect was transient, and transcripts levels fell to their basal levels by 4 days after treatment. The expression of these genes was obviously high and was sustained for a long time in pathogen-infected plants that were pre-treated with MoHrip1. Similar effects in plants have previously been reported [43], [54], [55]. These data demonstrated MoHrip1 could induce the expression of *PR* genes in rice and strengthen the systemic resistance of rice seedlings to the rice blast pathogen.

Plant defense in response to pathogen attack is regulated by a complex network of signaling pathways involving in three key signaling molecules: salicylic acid (SA), jasmonic acid (JA) and ethylene (Et). All of these signaling molecules are involved in what appear to be two major pathogen defense signaling pathways: a SA-dependent pathway and a JA/Et-dependent pathway [38]. In this paper, we observed the expression levels of five genes involved in SA- or JA/Et-mediated signal transduction in MoHrip1-treated rice (Figure 9). Three marker genes involved in the SA signal pathway were obviously elicited by the MoHrip1 protein on the second day after treatment. Meanwhile, the relative expression level of the gene *OsLox2* involved in the JA/Et signaling pathway was also strongly increased on the second day after inoculation; however, the expression of *OsAos2*, another marker gene in the JA/Et pathway, did not show the same expression pattern after the elicitor treatment. Taken together, these results demonstrated that the defense-related gene expression induced by the MoHrip1 elicitor might depend mainly on the SA-dependent signaling pathway. However, the exact signaling pathway involved in MoHrip1-mediated signal transduction is still unclear and needs to be further elucidated. Furthermore, some other elicitors, including harpin [43] and elicitin [56], have previously been reported to be similarly dependent on the SA signaling pathway. Nevertheless, the role of the SA signaling pathway in the rice defense system mediated by MoHrip1 remains to be studied further.

In conclusion, we report a novel elicitor named MoHrip1 from *M. oryzae*. The recombinant MoHrip1 protein could elicit the HR in tobacco leaves and induce the production of multiple signaling molecules (such as ROS and NO) as well as the expression PR genes to enhance the rice systemic resistance, demonstrating that MoHrip1 is a good candidate as a rice plant defense activation agent. Furthermore, activating defense responses with MoHrip1 could also be exploited as an alternative method, such as transgenic crops or biopesticides, for control of the rice blast fungus *M. oryzae*, replacing or reducing the application of chemical pesticides and thus benefitting human health. However, further research to elaborate the precise signaling pathway induced by MoHrip1 and some cell biology events involved in MoHrip1 and its receptor activation in rice must be undertaken before MoHrip1 can be applied in agriculture. Our studies will provide a novel paradigm for screening elicitor proteins from pathogenic fungi to strengthen resistance of their host plants against plant disease. The gene encoding MoHrip1 may be widely used in transgenic rice [57] and biopesticides in the future.

## Materials and Methods

### Pathogen and plant cultivation

*Magnaporthe oryzae* strain RO1-1 was originally isolated from diseased rice in Korea. The fungus was maintained on oat tomato agar (OTA) medium (30 g oatmeal, 150 ml tomato juice and 20 g agar in 1 L culture medium) at 28°C in the dark and cultured in yeast peptone dextrose (YPD) broth, which contained 1% yeast extract, 2% peptone and 2% dextrose, on a rotary shaker. Tobacco plants (*Nicotiana tabacum* cv. Samsun NN) were grown in a greenhouse at 24–26°C, with a 16-h light/8-h dark photoperiod, and rice cultivar Nipponbare (*Oryza sativa spp. japonica*) used in the study was also cultivated in the greenhouse under a 14-h-light/10-h-dark photoperiod at approximately 30°C during the day and approximately 25°C at night with 70–80% relative humidity [58].

### Establishment of tobacco cell culture

Tobacco seeds (*Nicotiana tabacum* cv. Samsun NN) were soaked in 75% ethanol for 30 sec, then in 10% sodium hypochlorite for 10 min, followed by 3 washes in sterilized water. The surface-sterilized seeds were cultivated for callus induction in Murashige and Skoog (MS) medium as previously described [59]. The calli were cut into small pieces after 15 days and suspended in liquid MS medium at pH 5.0 supplemented with inositol (100 mg/ml), 0.2% KH_2_PO_4_, 2,4-dichlorophenoxyacetic acid (0.2 mg/ml), thiamine hydrochloride (HCl, 1 mg/ml) and 3% sucrose in a shaker rotating at 130 rpm in the dark at 26°C.

### Elicitor bioassay

The HR-inducing activity of the proteins isolated at different purification steps was assayed in 8-week-old tobacco plants. The mesophyll tissue of fully developed leaves was infiltrated with samples (50 µl) or 20 mM Mes-NaOH buffer (control) using a syringe without a needle to cover areas of 1 cm^2^. The HR symptom necrosis was examined in the injected areas after 24 hours according to the previously described method [60]. Tobacco leaves with HR were stained by trypan blue and then were observed under a microscope, according to the previously described method [61].

### Protein preparation

*M. oryzae* strain RO1-1 was cultured in YPD broth at 25°C with shaking at 120 rpm for 15 days. The fungal culture was filtered through two layers of Whatman filter paper (0.45 µm, Millipore Corp., Billerica, MA, USA) and subjected to ammonium sulfate precipitation with 80% saturation overnight (on ice). The precipitate was resuspended in loading buffer (20 mM Mes-NaOH, pH 6.0) after centrifugation at 13,000×g for 45 min at 4°C and dialyzed (10000 Da molecular weight cut-off) overnight at 4°C. The concentrated fraction was collected and filtered with a 0.22 µm membrane (Millipore, Corp., Billerica, MA, USA). Further purification was performed with the AKTA Explorer 10 Protein Purification System (GE Healthcare, Piscataway, NJ, USA). Shortly afterwards, the concentrate was loaded on an anion exchange chromatography column (HP Q HiTrap™ 5 ml, GE Healthcare, Uppsala, Sweden), which had been pre-equilibrated with elution buffer (20 mM Mes-NaOH, pH 6.0). The adsorbed proteins were eluted with a linear NaCl gradient (0 to 1 M in elution buffer) at a flow rate of 3.5 ml/min, while the unbound material was removed with the pre-equilibration buffer. All fractions were collected, applied to a desalting column (GE Healthcare, Uppsala, Sweden) and tested for elicitor activity. The purified protein was monitored by its ability to induce HR in tobacco leaves. The fraction showing the greatest ability to induce HR was pooled and concentrated by ultrafiltration (Amicon Ultra-15, 10,000 MW cut-off, Millipore). The concentrated fraction was further purified with native-PAGE and retrieved with elution electrophoresis (Bio-Rad, Benicia, USA). Each protein fraction was purified using 15% SDS-PAGE and tested for elicitor activity. Finally, the active fractions were pooled and concentrated by ultrafiltration, washed with 20 mM Mes-NaOH, pH 6.0 buffer three times and stored at −20°C for subsequent use.

### Partial amino acid sequence identification

The protein sample was isolated by 2-DE and subjected to mass spectrometry analysis as described previously [62]. In short, the tryptic peptides resulting from in-gel digestion were loaded onto a C18 reversed-phase column (75 µm×8 cm) packed with 5–15 µm spherical particles (Grace Vydac, Columbia, MD, USA). The tip size of the analytical column was 2 µm, and the flow rate was 20–50 nL/min. To elute peptides from the column, a Waters 2695 Alliance High-Performance Liquid Chromatography (HPLC) System (Waters Corp., Milford, MA, USA) was used to generate the following gradient: 0–5% B in 5 min, 5–40% B in 25 min, 40–100% B in 10 min (A = 0.1 M acetic acid in water, B = 0.1 M acetic acid/70% acetonitrile).

MALDI-TOF and a quadrupole time-of-flight (Q-TOF) mass spectrometer (Thermo Electron Corp., San Jose, CA, USA) with a nano-electrospray ionization (ESI) ion source were used to evaluate the peptides resulting from protease digestion. The major peaks were further analyzed by de novo sequencing (National Center of Biomedical Analysis, Beijing, China). The resulting peptide map was analyzed by MASCOT peptide mass fingerprint software (Matrix Science, London; http://www.matrixscience.com).

### Gene cloning of *mohrip1*

The elicitor-encoding gene *mohrip1* was amplified from *M. oryzae* by RT-PCR. Total RNA was extracted using the E.Z.N.A.™ Fungal RNA Kit (Omega Bio-tek, Norcross, GA, USA), and cDNA was synthesized using a TransScript™ Two-Step RT-PCR Super Mix Kit (TransGen Biotech, Beijing. China) with oligo dT as primers. Based on the Basic Local Alignment Search Tool (BLAST) results of the peptide sequence in the National Center for Biotechnology Information database gained by MS and *de novo* sequencing, a pair of gene-specific primers was designed to amplify the entire coding sequence of the elicitor-encoding gene from *M. oryzae*. The primer sequences were designed as follows: forward primer, 5′-ATGCGCTTCGCCACCATCACCG-3′ and reverse primer, 5′-CTAAGCGGAGCCGTCAATGGCAATA-3′. The amplified and purified fragment was cloned into the pMD 18-T vector (TaKaRa Biotechnology, Dalian, China) and transformed into DH5α *E. coli* (TaKaRa Biotechnology, Dalian, China). The transformants were cultured in LB liquid medium in a rotary shaker for 12 h. The cells were harvested by centrifugation and used for extraction of the plasmid including the elicitor-encoding gene. The cDNA of *mohrip1* was verified by DNA sequencing.

### Heterologous expression and purification of recombinant protein

The *mohrip1* gene was inserted into the *BamH*1/*Sal*1 sites of the His-tagging pET-30a (+) vector (Novagen, USA), which was then transformed into the *E. coli* strain transetta (DE3) chemically competent cells (TransGen Biotech, Beijing, China) to express the elicitor as a C-terminally His_6_-tagged protein. The primers, which included the *BamH*1/*Sal*1 restriction sites and the 5′ and 3′ ends of the *mohrip1* gene, were designed as follows: forward primer, 5′- GGATCCATGCGCTTCGCCACCATCACCG-3′ (the *BamH*1 site is underlined) and reverse primer, 5′- GTCGACCTAAGCGGAGCCGTCAATGGCAATA-3′ (the *Sal*1 site is underlined), and the clones of the *MoHrip1* gene insertion were identified by PCR. The PCR program was as follows: 95°C for 4 min, followed by 35 cycles of 95°C for 30 s, 65°C for 30 s and 72°C for 30 s and a final extension at 72°C for 10 min. The DNA was isolated by electrophoresis in a 1.5% agarose gel and observed by staining with Gold View (SBS Genetech, Beijing, China) and using DL2000 as a DNA marker (TaKaRa Biotechnology, Dalian, China).

To express the recombinant MoHrip1 protein, the bacteria were first grown for 3 h, and then the recombinant protein was induced by the addition of 1 mM isopropyl β-D-1-thiogalactopyranoside (Sigma, St. Louis, MO, USA) to the media. Three hours after induction, the cells were collected by centrifugation and disrupted three times with an ultrasonic disruptor. The supernatant, including the recombinant protein, was concentrated through centrifugation at 15,000×g for 30 min. The purification of recombinant MoHrip1 mainly consisted of three sequential procedures as follows: affinity chromatography with a His-Trap HP column (GE Healthcare, Waukesha, WI, USA), ion-exchange chromatography with a Mono Q column (GE Healthcare, Waukesha, WI, USA) and size-exclusion chromatography with a Superdex-200 column (GE Healthcare, Waukesha, WI, USA). The purified protein was detected by SDS-PAGE and staining with Coomassie Brilliant Blue. A protein molecular marker (Fermentas, Glen Burnie, MD, USA) was used to evaluate the sizes of the purified recombinant proteins.

### Characteristics of the protein elicitor

To determine the minimum concentration of MoHrip1 (dissolved in sterile distilled water) for HR activity, serially diluted protein solutions of 0.01, 0.1, 0.5, 1, 5, 10 and 30 µM were infiltrated into tobacco leaves. The same concentration of bovine serum albumin was injected into tobacco leaves as negative controls.

The pI of the MoHrip1 protein was determined by 2-DE with immobilized pH gradients.

The Pierce® Glycoprotein Staining Kit (Thermo Scientific, Waltham, MA, USA) was used to determine whether MoHrip1 was a glycoprotein.

To determine elicitor heat stability, five aliquots of purified protein were incubated at 4, 25, 60, 80 and 100°C for 15 min, and then the HR activity of the treated proteins was tested.

Enzymatic digestion of the protein elicitor was performed as described [63]. MoHrip1 (5 µM) was incubated with protease K (100 µg/ml) for approximately 2 h at 37°C and then used for the HR bioassay.

### Detection of hydrogen peroxide production in tobacco leaves and cell culture

The histological localization of the H_2_O_2_ in tobacco leaves was observed as previously described [35], [64]. The 6-week-old tobacco leaves were infiltrated with MoHrip1 (20 µM) or Mes-NaOH as a control. At 24 h post-treatment, the leaves were cut, soaked in a 3,3′-diaminobenzidine (DAB)-HCl (1 mg/ml, pH 3.8) solution (Sigma, MO, USA) and incubated for 8 h prior to sampling in the dark. The leaves were then put into boiling ethanol (95%) for 20 min to remove the chlorophyll and subsequently stored in 70% glycerol. The H_2_O_2_ production in tobacco leaves was examined under a microscope.

H_2_O_2_ production in cell culture was detected by chemiluminescence, using luminol as a reagent [65]. Briefly, a 250 µl aliquot of the cell samples was added to 300 µl of a buffer containing 175 mM mannitol, 0.5 mM CaCl_2_, 0.5 mM K_2_SO_4_ and 10 mM HEPES, pH 5.75. After incubation for 1 h at 26°C on a rotary shaker (150 rpm), 10 µl MoHrip1 (5 µM) and 50 µl of 0.3 mM luminol were put into buffer, and the chemiluminescence, measured within a 30 s period with the GloMax®-96 Luminometer (Promega, Madison, WI, USA), was integrated and presented as nanomoles of H_2_O_2_ per gram fresh weight of suspended cells, using a standard calibration curve measured by adding of H_2_O_2_ to tobacco cell suspension aliquots.

### Analysis of callose deposition in tobacco leaves and extracellular culture medium pH in tobacco cell culture

To visualize callose deposition, two-month-old tobacco leaves were treated with the MoHrip1 protein and stained with aniline blue (Aladdin, Shanghai, China) at 24 h post-treatment, as described previously [66]. In brief, the leaves were fixed overnight in a solution of 1% (v/v) glutaraldehyde, 5 mM citric acid and 90 mM Na_2_HPO_4_ (pH 7.4). The chlorophyll was removed, and the samples were dehydrated in 100% ethanol. The transparent leaves were transferred sequentially into 50% (v/v) ethanol and equilibrated in 67 mM K_2_HPO_4_ (pH 12.0) before staining for 1 h at room temperature in 0.1% (w/v) aniline blue dissolved in 67 mM K_2_HPO_4_ (pH 12.0). The stained leaves were transferred onto a microscope slide in 70% (v/v) glycerol and 30% (v/v) staining solution and examined under an ultraviolet epifluorescence microscope (Carl Zeiss, Jena, Germany). The callose deposits were observed as pale-blue fluorescence.

To measure the alkalinization of the growth medium, the extracellular pH was measured directly in the medium of tobacco suspension cells [67]. The experiments were performed simultaneously in two 10 mL flasks (control and test), each containing 2 g fresh weight (f.wt) of cells per 5 ml of suspension medium, at 26°C, with orbital shaking at 120 rpm. For each replicate, the pH of medium at the start of the experiment was between 5.0 and 5.2. Simultaneous changes in pH were monitored in both suspension media with a pH meter (Sartorius Stedim, Germany) with pH-sensitive combined electrodes functioning in parallel. MoHrip1 was added when a stable pH was obtained, and the pH values were observed for 180 min after MoHrip1 addition.

### Bioassay for MoHrip1-induced disease resistance in rice

Sixteen rice seedlings were grown in a flowerpot, and at the three-leaf stage, Nipponbare rice plants (*Oryza sativa spp. japonica*) sprayed with either elicitor (5 µM) or Mes-NaOH (20 mM) as a negative control were tested for resistance against rice blast. After 3 days of incubation at 26°C and 70–80% relative humidity, detached plant leaves were sprayed with an aqueous suspension of 1×10^6^ *M. oryzae* spores per milliliter containing 0.05% (v/v) Tween 20 [68]. The inoculated plants were maintained at 26°C and 100% relative humidity in a dark chamber for 24 h and then transferred to a greenhouse at 26°C and 70–80% relative humidity under a 14-h-light/10-h-dark photoperiod. Leaf blast symptoms were investigated at 7 days post-inoculation when typical lesions appeared on the leaves of control rice plants. The disease indices of MoHrip1-treated and control plants infected with rice blast were compared. Each seedling was examined and rated on a scale of 0–9 (0 = resistant and 9 = susceptible) according to the international specification for rice blast disease.

### Assay of H_2_O_2_ production in rice leaves

Active oxygen species released by leaf tissues were assayed by peroxide-dependent luminescence of luminol. Leaves of 6-week-old rice plants removed from the growth chamber were sliced into approximately 1 mm strips and incubated in sterile distilled water in a 96-well plate for 12 h to allow wound injury-related ion leakage. Equal amounts of leaf tissues were then treated with 5 µM MoHrip1 (dissolved in sterile distilled water), 1 µM flg22 or sterile distilled water, in 200 µl of buffer concluding 1 µg horseradish peroxidase (HRP) and 20 µM luminol (Sigma-Aldrich, St. Louis, MO, USA). Luminescence was recorded for 20 min with a GloMax®-96 microplate Luminometer (Promega, Madison, WI, USA). Each data point represents at least three replicates.

### Analysis of the expression of pathogenesis-related genes induced by MoHrip1 using real-time RT-PCR

To investigate the mechanism of the defense responses induced by MoHrip1 in rice plants, the relative expression levels of two rice PR genes (*OsPR-1a* and *OsPR-10a*) [69], [70] and five signaling pathway-related genes (*OsEDS1*, *OsPAL1*, *OsNH1*, *OsLOX2* and *OsAOS2*) [71] were examined. Total RNA was extracted from *M. oryzae*-inoculated plants pretreated with MoHrip1 or not and uninoculated plants treated with MoHrip1 with the TRIzol reagent (Invitrogen, Carlsbad, CA, USA). cDNA was produced using the TransScript™ Two-Step RT-PCR Super Mix Kit (TransGen Biotech, Beijing, China), and the concentrations of the cDNAs were adjusted to be the same. The expression patterns of *PR* genes were analyzed with real-time PCR, and the *Osactin* gene was amplified as a quantitative control. The real-time RT-PCR was conducted using SYBR® Premix Ex Taq™ (TaKaRa Biotechnology, Dalian, China) and an iCycler MyiQ™2 (Bio-Rad, Hercules, CA, USA) according to the manufacturer's instructions. At least three independent biological samples were used with specific primers for each individual gene (Table 2). The data were normalized by the value of actin genes, the fold change in the expression level compared with healthy rice leaves was calculated, and standard deviation values are shown.

**Table 2:** Primers used for real-time RT-PCR of pathogenesis-related and actin genes.

Genes	Forward primer (5′-3′)	Reverse primer (5′-3′)	Accession numbers
*OsPR-1a*	GTATGCTATGCTACGTGTTTATGC	GCAAATACGGCTGACAGTACAG	AJ278436
*OsPR-10a*	GGCTTGGTCGACGACATTG	CAGGGTTAAGCTTCATGGTGTAGA	AF274850
*OsEDS1*	CCCCGCATACCACTTACT	TGTTGATGAAACCACTCCC	AK100117
*OsPAL1*	GGTGTTCTGCGAGGTGATGA	AGGGTGGTGCTTCAGCTTGT	AK068993
*OsNH1*	ATCTTGATGATGCGTTTGC	TCAGCTTGCTCCAGTATTTC	AK120715
*OsLOX2*	AGATGAGGCGCGTGATGAC	CATGGAAGTCGAGCATGAACA	AK241395
*OsAOS2*	TACCAGCCGTGCGCCACCAG	AGGACGGAGCTGGTTGAGTGG	AK061758
*Osactin*	GAGTATGATGAGTCGGGTCCAG	ACACCAACAATCCCAAACAGAG	AK060893

### Protein assay

The concentrations of all proteins used in this study were measured with the BCA™ Protein Assay Kit (Pierce, Rockford, IL, USA).

### Statistical analysis

All experiments and data provided in this paper were repeated at least three times. The data are presented as the means ± the standard deviation, and significant differences between the treatments and the controls were decided by analysis of variance using SAS.

## Supporting Information

Figure S1 **Two-dimensional gel electrophoresis analysis of the MoHrip1 elicitor.** MoHrip1 protein (250 µl) was loaded onto a 13 cm IPG strip with a linear gradient of pH 3–10 to perform separation in the first dimension. The secondary separation was performed by 15% SDS-PAGE, and the gel was then stained with Coomassie Brilliant Blue R-250. A pI of 4.53 was estimated based on the protein's relative location on the gel.
(TIF)

Figure S2 **Glycosyl-specific staining and Coomassie Brilliant Blue staining of purified protein.** Horseradish peroxidase was used as a positive control, and soybean trypsin was used as a negative control. **A**. Glycosyl-specific staining of SDS-PAGE, **B**. Coomassie Brilliant Blue stained SDS-PAGE. M: Protein molecular weight marker, 1: Positive control, 2: Negative control, 3: MoHrip1.
(TIF)

Figure S3 **Signal peptide analysis of the MoHrip1 elicitor using the SignalP 4.0 Server.** The signal peptide of MoHrip1 was predicted and underlined, which contains 16 amino acids, demonstrating that MoHrip1 elicitor is a secreted protein.
(TIF)
