# Supplementary figures and images for: Purification and Characterization of a Novel Hypersensitive Response-Inducing Elicitor from Magnaporthe oryzae that Triggers Defense Response in Rice
Source: PLoS One. 2012 May 18;7(5):e37654. doi: 10.1371/journal.pone.0037654 (PMC3356297; doi:10.1371/journal.pone.0037654)

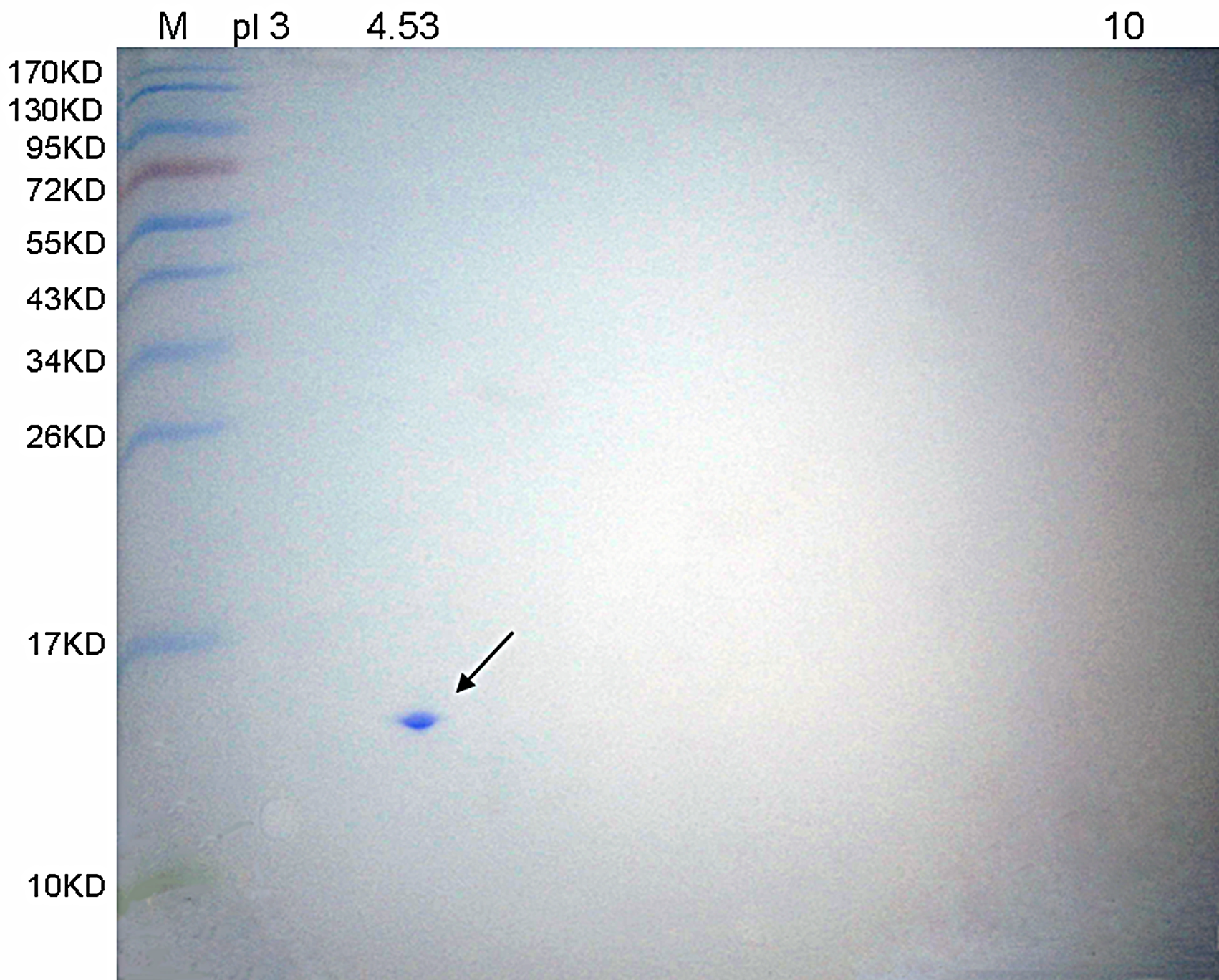

Supplement: Figure S1 — Two-dimensional gel electrophoresis analysis of the MoHrip1 elicitor. MoHrip1 protein (250 µl) was loaded onto a 13 cm IPG strip with a linear gradient of pH 3–10 to perform separation in the first dimension. The secondary separation was performed by 15% SDS-PAGE, and the gel was then stained with Coomassie Brilliant Blue R-250. A pI of 4.53 was estimated based on the protein's relative location on the gel. (TIF) [file pone.0037654.s001.tif]

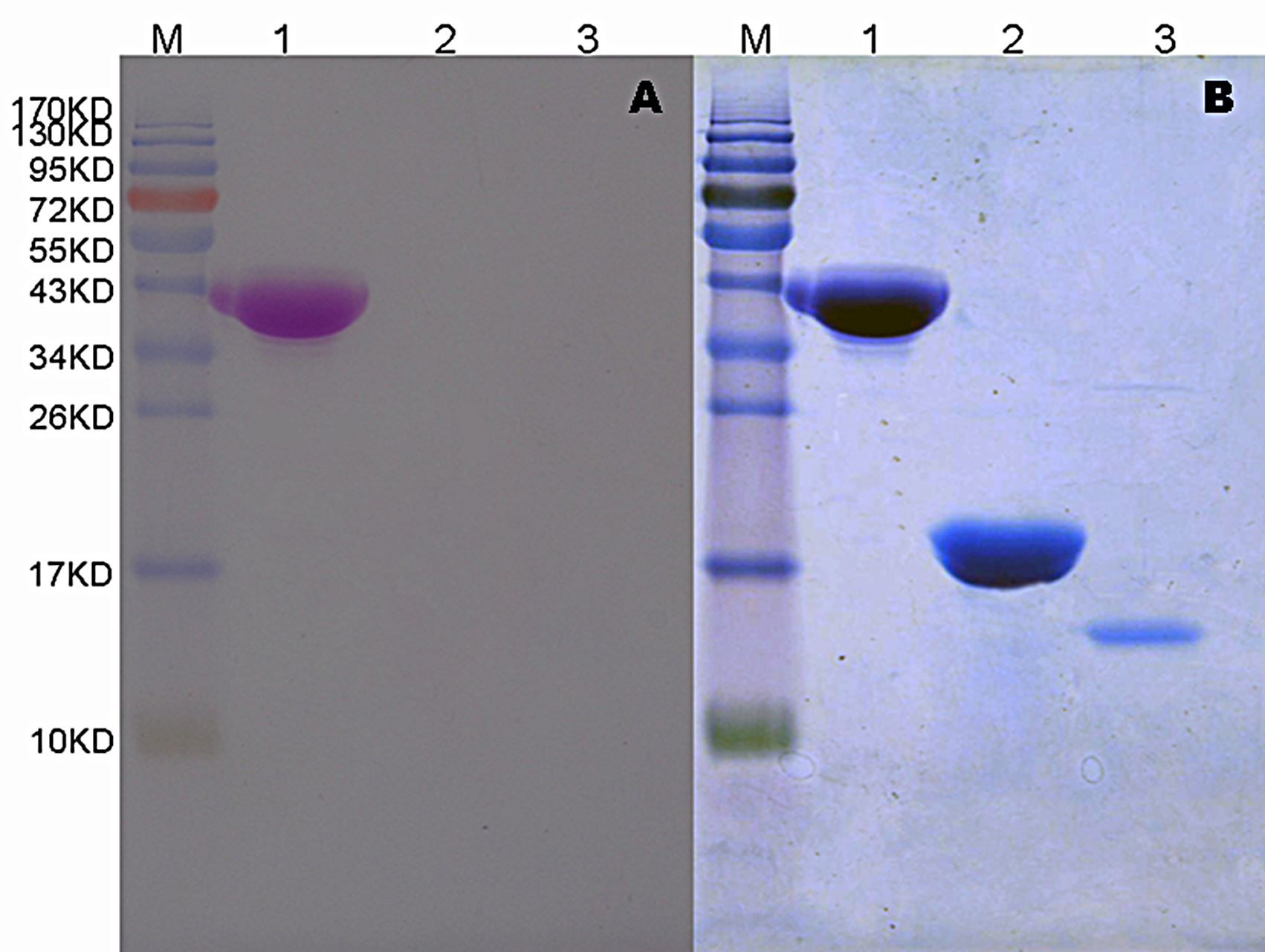

Supplement: Figure S2 — Glycosyl-specific staining and Coomassie Brilliant Blue staining of purified protein. Horseradish peroxidase was used as a positive control, and soybean trypsin was used as a negative control. A. Glycosyl-specific staining of SDS-PAGE, B. Coomassie Brilliant Blue stained SDS-PAGE. M: Protein molecular weight marker, 1: Positive control, 2: Negative control, 3: MoHrip1. (TIF) [file pone.0037654.s002.tif]

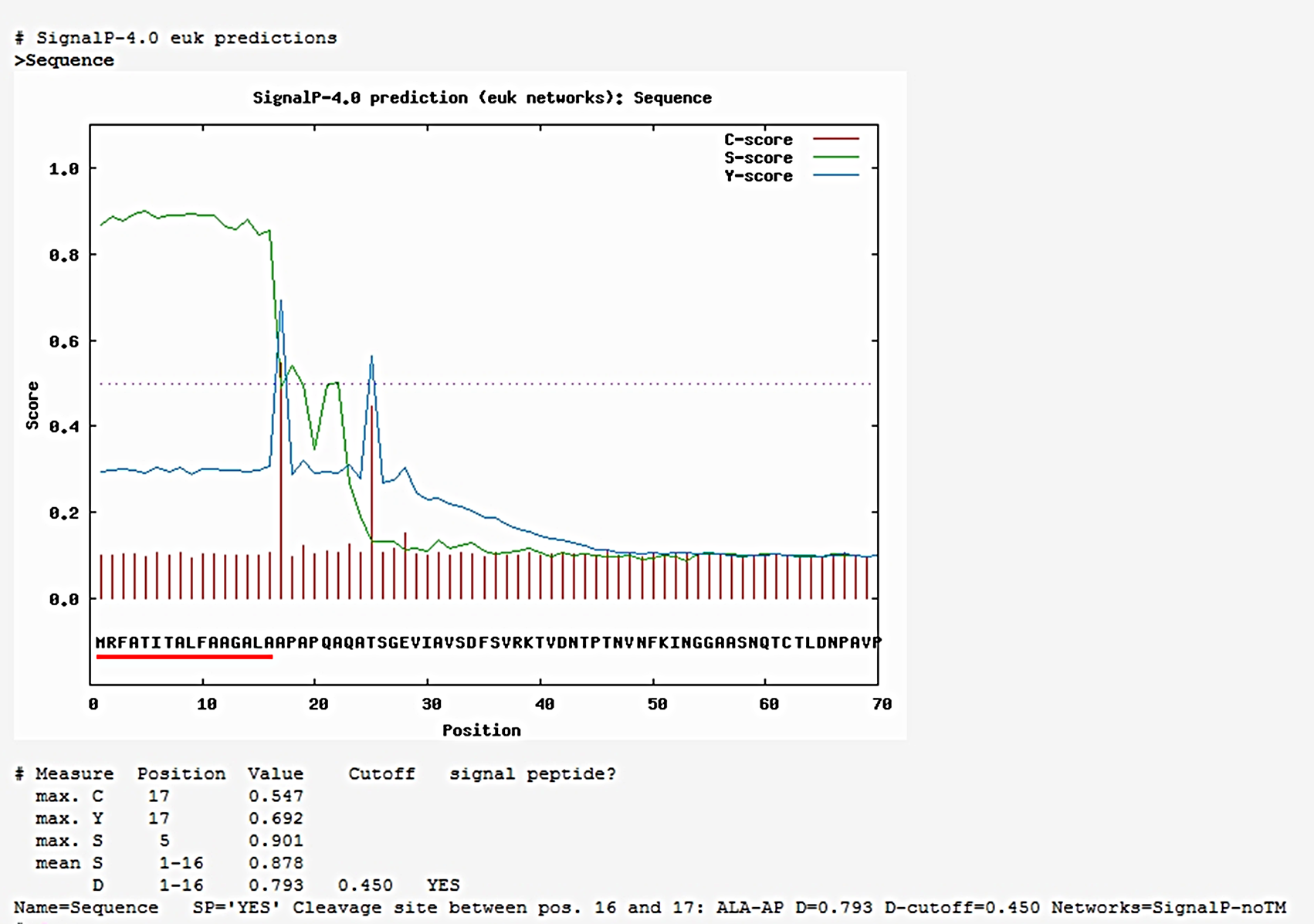

Supplement: Figure S3 — Signal peptide analysis of the MoHrip1 elicitor using the SignalP 4.0 Server. The signal peptide of MoHrip1 was predicted and underlined, which contains 16 amino acids, demonstrating that MoHrip1 elicitor is a secreted protein. (TIF) [file pone.0037654.s003.tif]
